# Dual-time-point F-18 FDG PET/CT imaging for differentiating the lymph nodes between malignant lymphoma and benign lesions

**DOI:** 10.1007/s12149-012-0669-1

**Published:** 2012-11-28

**Authors:** Michihiro Nakayama, Atsutaka Okizaki, Shunta Ishitoya, Miki Sakaguchi, Junichi Sato, Tamio Aburano

**Affiliations:** 1 Department of Radiology, Asahikawa Medical University, 2-1-1-1 Midorigaoka-higashi, Asahikawa, 078-8510 Japan; 2 Division of Radiology, Asahikawa Medical University Hospital, Asahikawa, Japan

**Keywords:** 18F-FDG PET/CT, Dual-time-point imaging, Lymphoma, Benign lymph node, Standardized uptake value

## Abstract

**Purpose:**

The purpose of the present study is to evaluate the clinical value of dual-time-point F-18 FDG PET/CT imaging to differentiate malignant lymphoma (ML) from benign lymph node (BLN).

**Materials and methods:**

The subjects were 310 lymph nodes in 84 patients (195 ML lesions in 30 patients and 115 BLN in 54 patients associated with various etiologies.). F-18 FDG PET/CT scan was performed at 50 min (early scan) and at 100 min (delayed scan) after the injection. First, the maximum standardized uptake value (SUVmax) of each lesion at early and delayed scans was calculated. Second, we estimated the difference between early and delayed SUVmax (D-SUVmax) and the retention index (RI-SUVmax) to evaluate the change of tracers in the lesions. Furthermore, proper cut-off values of them were evaluated using receiver operating characteristic analysis. The efficacy of each parameter was analyzed with ANOVA.

**Results:**

Delayed SUVmax and D-SUVmax in ML were significantly higher than those in BLN. Proper cut-off value in delayed SUVmax was 4.0 and in D-SUVmax was 1.0. When the proper cut-off value in D-SUVmax was applied, the D-SUVmax yielded the role of diagnosis with sensitivity of 82.6 %, specificity of 65.2 %, positive predictive value of 80.1 % and negative predictive value of 68.8 %, respectively.

**Conclusions:**

The delayed SUVmax and D-SUVmax were useful indices to differentiate ML from BLN, regardless of histologic subtype. Dual-time-point F-18 FDG PET/CT imaging may help to consider whether there is any need to proceed to more invasive tests, such as biopsy, in individual patients.

## Introduction

Malignant lymphoma (ML) is one of the most common hematologic malignancy [1]. According to the National Cancer Institute in the US, there were 75,190 new cases of the malignant lymphomas in the US in 2011, and 20,620 deaths from the diseases. Fluorine-18-fluorodeoxyglucose (F-18 FDG) positron emission tomography/computed tomography (PET/CT) is widely used for staging and treatment evaluation of ML [2, 3]. PET/CT offers advantages over conventional imaging techniques, because it can demonstrate abnormal metabolic activity and extension of malignant diseases [4–6]. However, many inflammatory lesions such as pneumonia, sarcoidosis, rheumatoid arthritis, etc., also have elevated F-18 FDG uptake in PET, leading to false-positive results [7–10]. Moreover, there is considerable overlap between the maximum standardized uptake values (SUVs) of ML and BLN, causing difficulty in correctly diagnosing F-18 FDG PET data [11–13]. Some reports indicated that F-18 FDG PET was sensitive and specific in the diagnosis and staging for patients with ML on single-time-point scans [14, 15]. In patients with head and neck malignant tumors [16], pulmonary nodules [17, 18], breast cancer [19], pancreas tumors [20], and bone malignant tumor [21], several authors have reported significant improvement in the diagnostic accuracy of FDG PET scan with dual-time-point scans.

F-18 FDG uptakes in many malignant lesions are increased with time interval [22, 23]. In patients with ML, recently, Shinya et al. [2] statistically evaluated the differences of FDG uptake in early and delayed scans, and compared the maximum SUV (SUVmax) and the retention index (RI) of SUVmax (RI-SUVmax) between the different grades of lymphoma. Because various cell types exhibit varying rates of F-18 FDG uptake [16, 17, 19, 23, 24], we hypothesized that measurements of SUVmax at two time points may prove to be of value in differentiating ML from BLN.

Therefore, the purpose of this study was to assess the usefulness of dual-time-point F-18 FDG PET/CT scan in initial diagnosis of ML, with analyses of the SUVmax on 50-min early and 100-min delayed scans.

## Materials and methods

### Patient population

The subjects consisted of 30 patients (15 men and 15 women; mean age, 64.6 ± 12.2 years) with 195 lymph nodes of ML and 54 patients (26 men and 28 women; mean age, 61.7 ± 17.7 years) with 115 benign lymph nodes associated with various etiologies. Patient characteristics are summarized in Table 1. All patients underwent F-18 FDG PET/CT scanning for the differential diagnosis from July 2009 to May 2011. They were finally diagnosed according to clinical and radiologic follow-up (follow-up period of time; at least 6 months) and/or histopathological findings. The histopathology examinations confirmed 30 patients with ML and 29 patients with BLN. 25 patients with BLN were finally diagnosed according to clinical and radiologic follow-up (8.74 ± 3.30 months, range 6–18 months). Reactive lymph nodes are defined as lymph nodes, which diagnosed no malignancy through a clinical follow-up with no treatment or proof of no increase in size and accumulations on F-18 FDG PET/CT scans, in the patients with suspected malignancy. None of the patients had received chemotherapy or radiation therapy before the dual-time-point F-18 FDG PET scans. Informed consent was obtained from all patients participating in the study. This study was retrospective and ethics committee approval was deemed unnecessary in our institution.

**Table 1:** Characteristics of malignant lymphoma and benign lymph nodes

	Number of patients	Number of lesions	Early SUVmax (mean ± SD)	Delayed SUVmax (mean ± SD)	D-SUVmax (mean ± SD)	RI-SUVmax (mean ± SD)
*Malignant lymphoma* ^a^						
Histological subtypes of lymphoma						
Diffuse large B-cell lymphoma	12	74	7.24 ± 6.18	9.35 ± 7.05	2.11 ± 1.41	0.42 ± 0.24
Follicular lymphoma	10	70	8.13 ± 5.75	10.18 ± 6.80	2.05 ± 1.56	0.32 ± 0.23
T-cell lymphoma	5	34	4.19 ± 2.58	5.79 ± 3.01	1.60 ± 0.87	0.43 ± 0.19
Hodgkin’s lymphoma	2	13	6.44 ± 3.57	8.06 ± 3.67	1.62 ± 0.67	0.30 ± 0.13
Mantle cell lymphoma	1	4	4.18 ± 0.62	5.28 ± 0.66	1.10 ± 0.19	0.27 ± 0.07
Total	30	195	6.70 ± 5.43	8.62 ± 6.27	1.91 ± 1.33	0.38 ± 0.23
*Benign lymph nodes* ^b^						
Disorders						
Reactive lymph node	24	54	3.03 ± 0.90	3.69 ± 1.02	0.65 ± 0.52	0.23 ± 0.20
Lymphadenitis	10	20	3.88 ± 3.06	5.34 ± 4.04	1.47 ± 2.00	0.34 ± 0.40
Pneumonia	9	16	2 .88 ± 0 .74	3 .71 ± 1.09	0.82 ± 0.63	0.29 ± 0.20
Rheumatoid arthritis	3	9	3.26 ± 1.32	3.62 ± 1.15	0.37 ± 0.31	0.16 ± 0.16
Sarcoidosis	2	5	7.60 ± 4.37	8.26 ± 4.80	0.66 ± 0.89	0.09 ± 0.10
Silicosis	1	5	2 .60 ± 0 .39	3.66 ± 0.72	1.06 ± 0.60	0.41 ± 0.27
IgG4-related disease	1	2	3. 50 ± 1. 00	3.80 ± 1.30	0.30 ± 0.30	0.07 ± 0.07
Tuberculosis	1	1	2.60	3.20	0.60	0.23
Echinococcus	1	1	4.50	5.60	1.10	0.24
Benign lung tumor	1	1	1.50	1.90	0.40	0.27
Benign brain tumor	1	1	4.40	4.80	0.4	0.09
Total	54	115	3.37 ± 2.43	4.16 ± 2.44	0.80 ± 1.04	0.25 ± 0.25

^a^Size of lesions: 1.2 cm ± 0.8 in short-axis diameter, range 0.3–2.7 cm

^b^Size of lesions: 1.0 cm ± 0.5 in short-axis diameter, range 0.3–2.4 cm

### F-18 FDG PET/CT scans

All imaging and data acquisition was performed on a combined PET/CT in-line system (Discovery VCT, GE Healthcare, Milwaukee, WI, USA). This device integrates a PET scanner with a 64 multi-detector row CT and permits the acquisition of coregistered CT and PET images in the same session. At the time of F-18 FDG injection, all patients had fasted for at least 4 h and had blood sugar levels of less than 120 mg/dl. The scan was performed twice: an early whole-body (from head to pelvis) scan at 50 min after the injection of 3.7 MBq/kg of F-18 FDG, followed by a delayed scan at 100 min. The scans consisted of 7 or 8 beds, and the length of one bed was 16 cm. A delayed scan was performed for the same field of view and scan area in early scan. The image acquisition time per bed was 2–3 min. The PET images were reconstructed iteratively with three dimensional ordered-subset expectation maximization (VUE point, GE Healthcare, Milwaukee, WI, USA). An unenhanced CT scan (100 mA with auto mA, 0.6 s/rotation, 120 kV) from the head to the pelvic floor was acquired (reconstructed slice thickness 3.27 mm) for fusing with PET images. PET/CT fusion images on three orthogonal (transaxial, coronal and sagittal) planes were reviewed on a workstation (Advantage Workstation version 4.4, GE Healthcare, Milwaukee, WI, USA).

### Image analysis

F-18 FDG accumulations were considered positive when focal uptake was more intense than the mediastinal blood-pool activity in the early scan or delayed scan. PET images were interpreted independently and prospectively by two experienced nuclear medicine physicians, without the knowledge of histopathologic or other radiologic data. In case of a discrepant reading, the two physicians discussed the case together to reach consensus about referral. The results of FDG uptake were classified by a four-score visual scale using the individuals’ blood-pool radioactivity as a reference. The classification was defined as follows: 0 radioactivity lower than that of blood-pool; 1 radioactivity equal to that of blood-pool; 2 radioactivity mild higher than that of blood-pool; and 3 radioactivity obviously higher than that of blood-pool. The short-axis diameter of lymph nodes was measured on CT scan. The maximum standardized uptake value (SUVmax) of lymph node-related F-18 FDG accumulation on 50 min (early SUVmax) and 100 min (delayed SUVmax) images after F-18 FDG injection was calculated as follows:

$$ {\text{SUV}} = \{ {\text{Tissue activity }}({\text{Bq}}/{\text{g}})\} /\{ {\text{Injected F}} - 18{\text{ FDG dose}} ({\text{Bq}})/{\text{body weight (g)}}\} $$

A region of interest (ROI) which was manually drawn for SUVmax calculation on early PET scans was placed over the area of maximal metabolic activity on the transaxial slice showing tumor-related increased uptake. On delayed PET scans, ROIs were placed in identical positions. In addition to RI-SUVmax, we tried to evaluate an usefulness of difference between early SUVmax and delayed SUVmax (D-SUVmax) in this study, as a more simple index. The RI-SUVmax and the D-SUVmax were calculated as follows:

$$ \begin{aligned} {\text{RI-SUVmax }} & = {\text{ (Delayed SUVmax}} - {\text{Early SUVmax)}}/{\text{Early SUVmax}} \\ {\text{D-SUVmax }} & = {\text{ Delayed SUVmax}} - {\text{Early SUVmax}} \\ \end{aligned} $$

We estimated these quantitative values to evaluate the change of tracers in the lesions at 50 and 100 min after the F-18 FDG injection.

All lymph nodes showed increased accumulations of F-18 FDG on early and delayed scans. D-SUVmax and RI-SUVmax were calculated with uptake of same lymph nodes on the both scans.

### Statistical analysis

Differences in visual scores analyzed with the Wilcoxon signed-rank test, using the STATISTICA 03J (StatSoft Inc., Tulsa, OK, USA). All data were expressed as mean ± standard deviation (SD). Receiver operating characteristic (ROC) analysis was performed to determine the proper cut-off value for the difference ML and BLN. The overall statistically difference of area under the curves (AUCs) was evaluated with ANOVA, and we compared differences between AUCs of those indices using DBM-MRMC, version 2.2, software *(* http://perception.radiology.uiowa.edu *)*. A paired 2-tailed *t* test was used to compare differences between ML and BLN in those indices. *P* values of less than 0.05 were considered statistically significant.

## Results

### ROC analysis

**Fig. 1:**
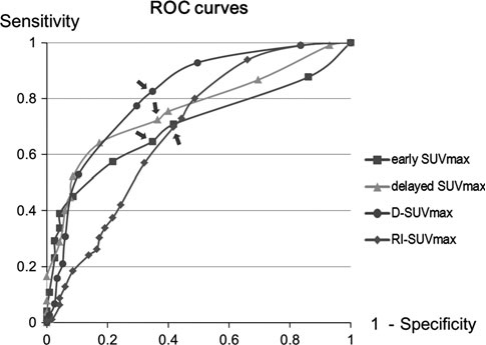
ROC analysis showed that the D-SUVmax provided a best statistical differentiation between ML and BLN. ROC analysis revealed that the proper cut-off values of early, delayed, D- and RI-SUVmax were 3.0, 4.0, 1.0 and 0.22, respectively (*arrows*)

The detailed data are described in Table 2. ROC analysis revealed that the use of delayed SUVmax and D-SUVmax provided better differentiation between ML and BLN than the use of early SUVmax and RI-SUVmax alone (Fig. 1). The AUCs in delayed SUVmax and D-SUVmax were statistically greater than in early SUVmax (*P* < 0.005). There was no significant difference between the AUCs in delayed SUVmax and D-SUVmax. The largest AUC has obtained with D-SUVmax in the four indices. The proper cut-off value of D-SUVmax for the differential diagnosis was >1.0; 161 of 195 ML and 75 of 115 BLN were correctly diagnosed using the cut-off value, yielding a sensitivity of 82.6 %, specificity of 65.2 %, positive predictive value (PPV) of 80.1 %, and negative predictive value (NPV) of 68.8 % (Table 2). The accuracy of D-SUVmax was 76.1 %, and the highest value compared with other indices or its combination.

**Table 2:** Diagnostic values for differentiation of ML and BLN with parameters of dual-time-point PET/CT scan

	Cut-off value	Sensitivity/specificity (%)	PPV/NPV (%)	AUC
Early SUVmax	3.0	70.8/58.2	74.2/56.8	0.715
Delayed SUVmax	4.0	75.4/60.0	76.2/61.0	0.772*
D-SUVmax	1.0	82.6/65.2	80.1/68.8	0.809*
RI-SUVmax	0.22	72.8/55.7	73.6/58.6	0.684

*PPV* positive predictive value, *NPV* negative predictive value, *AUC* area under the curve

* *P* < 0.005

### SUVmax and RI-SUVmax

**Fig. 2:**
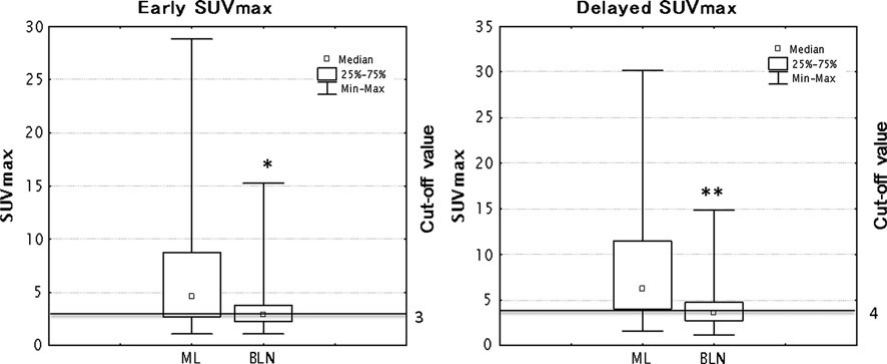
Box and whiskers plots showing distribution of early and delayed SUVmax among ML and BLN. The *graph* showed the distribution of data based on the five number summary: minimum, first quartile, median, third quartile, and maximum (*P < 0.01, compared with early SUVmax in ML, **P < 0.01, compared with delayed SUVmax in ML)

**Fig. 3:**
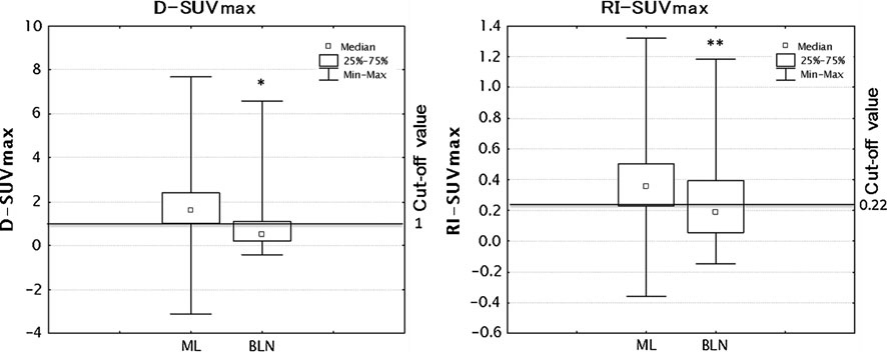
Box and whiskers plots showing distribution of D-SUVmax and RI-SUVmax among ML and BLN. The *graph* showed the distribution of data as mentioned in Fig. 2 (*P < 0.01, compared with D-SUVmax in ML, **P < 0.01, compared with RI-SUVmax in ML)

The mean values of early SUV max, delayed SUVmax, D-SUVmax, and RI-SUVmax were 6.70 ± 5.43, 8.62 ± 6.27, 1.91 ± 1.33 and 0.38 ± 0.23 in ML, and 3.37 ± 2.43, 4.16 ± 2.44, 0.80 ± 1.04 and 0.25 ± 0.25 in BLN, respectively. There were significant differences between ML and BLN in those indices (*P* < 0.01 for all comparisons; Figs. 2, 3). ML and BLN tended to demonstrate increase uptake in visual scores on delayed scans, but there was no significant difference between ML and BLN (Table 3).

**Table 3:** Visual scoring results: ML versus BLN on dual-time-point scan

Visual score	ML (early scan)	BLN (early scan)	ML (delayed scan)	BLN (delayed scan)
0	20	16	18	9
1	38	32	33	42
2	118	57	120	54
3	19	10	24	19
Total	195	115	195	115

## Discussion

The present study evaluated the usefulness of SUVmax and RI-SUVmax on dual-time-point F-18 FDG PET/CT scans, performed at 50 and 100 min after tracer injection, in patients with ML and BLN. The AUCs of delayed SUVmax and D-SUVmax were statistically greater than that of early SUVmax. D-SUVmax had the largest AUC among the four indices, which led to improved sensitivity and specificity.

The F-18 FDG uptake by lymphoma is probably associated with Glut1 expression [25, 26] and Ki-67 values [27, 28]. In many malignant tumors, scan start times of 45–60 min have been reported to cause significant underestimation of the true SUVs, because SUVs do not reach maximum levels until several hours after F-18 FDG injection [2, 23, 29]. Kumar et al. reported that F-18 FDG uptake in malignant cells was related to low glucose-6-phosphatase activity, and increased glucose uptake through glucose transporter proteins in these cells. In contrast, such a prolonged period of F-18 FDG uptake is rare in benign lesions or normal tissues [22, 23]. Gupta et al. [30] reported that the influx rate constant was greater in malignant lesions than in benign lesions, and that continuous tracer uptake by malignant lesions was observed in pulmonary tumor.

According to our results, delayed SUVmax and D-SUVmax were significantly better predictors of ML than early SUVmax. The AUC of D-SUVmax was the greatest among these four indices. Dual-time-point scans may have an advantage to observe the serial change of uptake in the lesions. Contrary to expectations, the present study showed that there was no significant difference in AUC between RI-SUVmax and early SUVmax. In principle, RI-SUVmax must have a merit of not being dependent on scale and is a useful index [2, 31]. Some authors reported RI is a good predictor for diagnosis and prognosis, and is superior to only early imaging [32, 33]. The reason is unknown why RI-SUVmax was not the best predictor, but our results are consistent with that of a previous study [18].

Some institutions, which give a high priority to economic requirements and protection of excessive radiation exposure, may perform only a single scan. In the *t* test to compare differences between ML and BLN, there was a significant difference between ML and BLN in all indices. According to this result, it may be preferable to use the early SUVmax cut-off value to avoid increased radiation exposure. However, obtaining these dual-time-point scans might have advantages such as discrimination of mesenteric lymph nodes from physiological intestinal accumulations [34] and discrimination of malignant tumors from benign tumors [17–22]. Focal FDG foci due to urinary excretion in the ureter can be mistaken as lymphadenopathy close to the ureter, such as ovary, cervix, pancreatic tail and adrenal grand [9, 35]. Co-registration of PET with anatomical imaging may be useful to avoid misinterpretation; however, it is still difficult to differentiate lesions in proximity to urinary activity, especially in a PET/CT study acquired without an intravenous contrast agent. In contrast, additional delayed scanning is a simple option [36]. In addition, the use of delayed SUVmax and D-SUVmax might improve the sensitivity and specificity for diagnosis of ML.

This study had several limitations. First, the subject population comprised a relatively small number of patients. The smallest group had only one patient, and the small numbers of subjects disturbed the analysis of differences in each histological subtype. Consequently, we performed ROC analysis without comparing each histological subtype. However, some authors have reported that the SUVmax tended to be lower in Hodgkin’s lymphoma and T-cell lymph nodes than in B-cell lymphoma, with differences observed in the early SUVmax [28]. Examination of a larger number of patients in a prospective study could help in evaluating the role of dual-time-point PET/CT scans in diagnosis of lymphoma. Second, the cut-off value obtained in this study was based on the data collected at our institution alone, and the absolute value of SUVmax may vary according to the imaging system used. We intend to address this issue by conducting similar comparisons at multiple institutions in future. Finally, histopathological examination of biopsy samples from all lymph nodes suspected of having F-18 FDG uptake on PET/CT was not performed. However, despite these limitations, our results suggest that the use of dual-time-point PET/CT scans might improve diagnostic capability for lymphoma compared with conventional techniques.

## Conclusion

In conclusion, dual-time-point F-18 FDG PET/CT scan is useful for differential diagnosis between ML and BLN, regardless of a variety of the histologic subtype. Dual-time-point F-18 FDG PET/CT imaging may help to consider whether there is any need to proceed to more invasive tests, such as biopsy, in individual patients.
